# Mosquito dynamics and their drivers in peri-urban Antananarivo, Madagascar: insights from a longitudinal multi-host single-site survey

**DOI:** 10.1186/s13071-024-06393-4

**Published:** 2024-09-10

**Authors:** Michaël Luciano Tantely, Hélène Guis, Manou Rominah Raharinirina, Maminirina Fidelis Ambinintsoa, Iavonirina Randriananjantenaina, Haja Johnson Velonirina, Christophe Revillion, Vincent Herbreteau, Annelise Tran, Romain Girod

**Affiliations:** 1 Unité d’entomologie médicale, Institut Pasteur de Madagascar, Antananarivo, Madagascar; 2 CIRAD–UMR ASTRE, Antananarivo, Madagascar; 3 Unité d’épidémiologie et de recherche clinique, Institut Pasteur de Madagascar, Antananarivo, Madagascar; 4 ASTRE, CIRAD, INRAE, Université de Montpellier, Montpellier, France; 5 Département d’Entomologie, Université d’Antananarivo, Antananarivo, Madagascar; 6 UMR ESPACE-DEV, Université de La Réunion, Saint-Pierre, La Réunion France; 7 IRD, UMR ESPACE-DEV, Phnom Penh, Cambodia; 8 CIRAD–UMR ASTRE, Montpellier, France; 9 CIRAD–UMR TETIS, Montpellier, France; 10 TETIS,Université de Montpellier, AgroParisTech, CIRAD, CNRS, INRAE, Montpellier, France

**Keywords:** Mosquito vectors, Mosquito-borne diseases, Climatic and environmental drivers, Peri-urban area, Single-site model, Madagascar

## Abstract

**Background:**

Antananarivo, the capital city of Madagascar, is experiencing a steady increase in population growth. Due to the abundance of mosquito vectors in this locality, the population exposed to mosquito-borne diseases is therefore also increasing, as is the risk of epidemic episodes. The aim of the present study was to assess, in a resource-limited setting, the information on mosquito population dynamics and disease transmission risk that can be provided through a longitudinal entomological study carried out in a multi-host single site.

**Methods:**

Mosquitoes were collected every 15 days over 16 months (from January 2017 to April 2018) using six CDC-light traps in a peri-urban area of Antananarivo. Multivariable generalised linear models were developed using indoor and outdoor densities of the predominant mosquito species as response variables and moon illumination, environmental data and climatic data as the explanatory variables.

**Results:**

Overall, 46,737 mosquitoes belonging to at least 20 species were collected, of which *Culex antennatus* (68.9%), *Culex quinquefasciatus* (19.8%), *Culex poicilipes* (3.7%) and *Anopheles gambiae* sensu lato (2.3%) were the most abundant species. Mosquito densities were observed to be driven by moon illumination and climatic factors interacting at different lag periods. The outdoor models demonstrated biweekly and seasonal patterns of mosquito densities, while the indoor models demonstrated only a seasonal pattern.

**Conclusions:**

An important diversity of mosquitoes exists in the peri-urban area of Antananarivo. Some well-known vector species, such as *Cx. antennatus*, a major vector of West Nile virus (WNV) and Rift-Valley fever virus (RVFV), *Cx. quinquefasciatus*, a major vector of WNV, *Cx. poicilipes*, a candidate vector of RVFV and *An. gambiae* sensu lato, a major vector of *Plasmodium* spp., are abundant. Importantly, these four mosquito species are present all year round, even though their abundance declines during the cold dry season, with the exception of *Cx. quinquefasciatus*. The main drivers of their abundance were found to be temperature, relative humidity and precipitation, as well as—for outdoor abundance only—moon illumination. Identifying these drivers is a first step towards the development of pathogen transmission models (R0 models), which are key to inform public health stakeholders on the periods of most risk for vector-borne diseases.

**Graphical Abstract:**

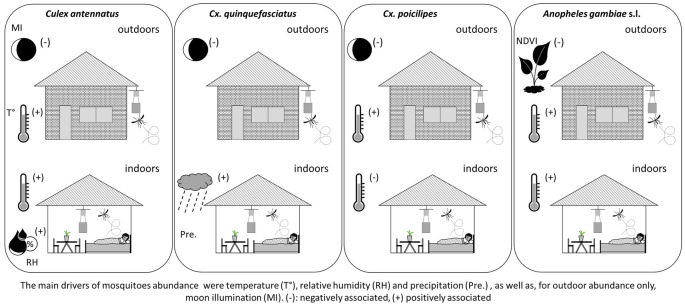

**Supplementary Information:**

The online version contains supplementary material available at 10.1186/s13071-024-06393-4.

## Background

In Madagascar, the pathogens that cause mosquito-borne diseases (MBDs) include haemosporidian parasites (*Plasmodium* spp., *Haemoproteus* spp.), parasitic nematodes (*Wuchereria bancrofti*) and arboviruses [1–3]. These pathogens are transmitted by 37 mosquito species (Additional file 1: Table S1) [1, 2, 4, 5, 6, 7, 8]. Mosquitoes are considered to be major vectors of pathogens when the following three criteria are fulfilled: (i) natural infection is highlighted in the field; (ii) vector competence is demonstrated in the laboratory; and (iii) vector-host contact is present. Mosquitoes that fulfil two criteria of these criteria are considered to be candidate vectors, and those that fulfil only one criterion are potential vectors [9]. The abundance and the behaviour of these vectors are key drivers of MBD transmission [10], and these characteristics are influenced by environmental and climatic factors in urban, rural or forested areas [11, 12].

In Madagascar, MBDs are mostly confined to rural and forested areas [1, 13]. However, a number of efficient mosquito vectors (*Anopheles gambiae* sensu lato [*A. gambiae* s.l.], *Culex antennatus* and *Culex quinquefasciatus*) [1, 14] and some of the pathogens they transmit (*Plasmodium* spp., Rift Valley fever virus [RVFV] and West Nile virus [WNV]) [1, 15, 16] occur in the urban area of Antananarivo, the capital city of Madagascar.

 Antananarivo is experiencing a high population growth rate (5%) [17], making it important to assess the risk of MBDs in its environment. Given the presence of pathogens and competent vectors, the human population growth in Antananarivo may increase the risk of city dwellers contracting MBDs [18, 19]. While a large number of studies have sought to detect the presence and to quantify the abundance of mosquito species known to be efficient vectors in Madagascar [1, 20], far fewer have investigated the drivers of their presence and abundance, especially in Antananarivo.

Studies identifying drivers of mosquito dynamics usually include multiple sampling sites [21, 22], with the aim to consider differences between sites, increase the precision and the power of the study and—depending on how the sites were selected—ensure representativity of the sampled areas. Yet, as studying mosquito dynamics implies repeated sampling, human and financial resources often limit the number of sites that can be included in any one survey. In the present study, we addressed the question of whether adequate information on mosquito dynamics and their drivers can be obtained in a longitudinal survey carried out in a single site, in a resource-limited setting.

Excluding several non-vector transmission risks of MBDs which are related to climate change listed in the literature [23], this study aims to assess whether the dynamics of vector abundance and its drivers can be characterised in a single site of a peri-urban area close to the capital city Antananarivo. A 16-month longitudinal study consisting of a series of mosquito catches was conducted at 2-week intervals on a multi-host farm. In particular, the aim was to assess if this sample effort was sufficient to: (i) inventorise mosquito species diversity; (ii) assess temporal variation in mosquito diversity and abundance; and (iii) develop statistical models to identify factors driving the variation of the abundance of mosquito species.

## Methods

### Mosquito sampling

**Fig. 1:**
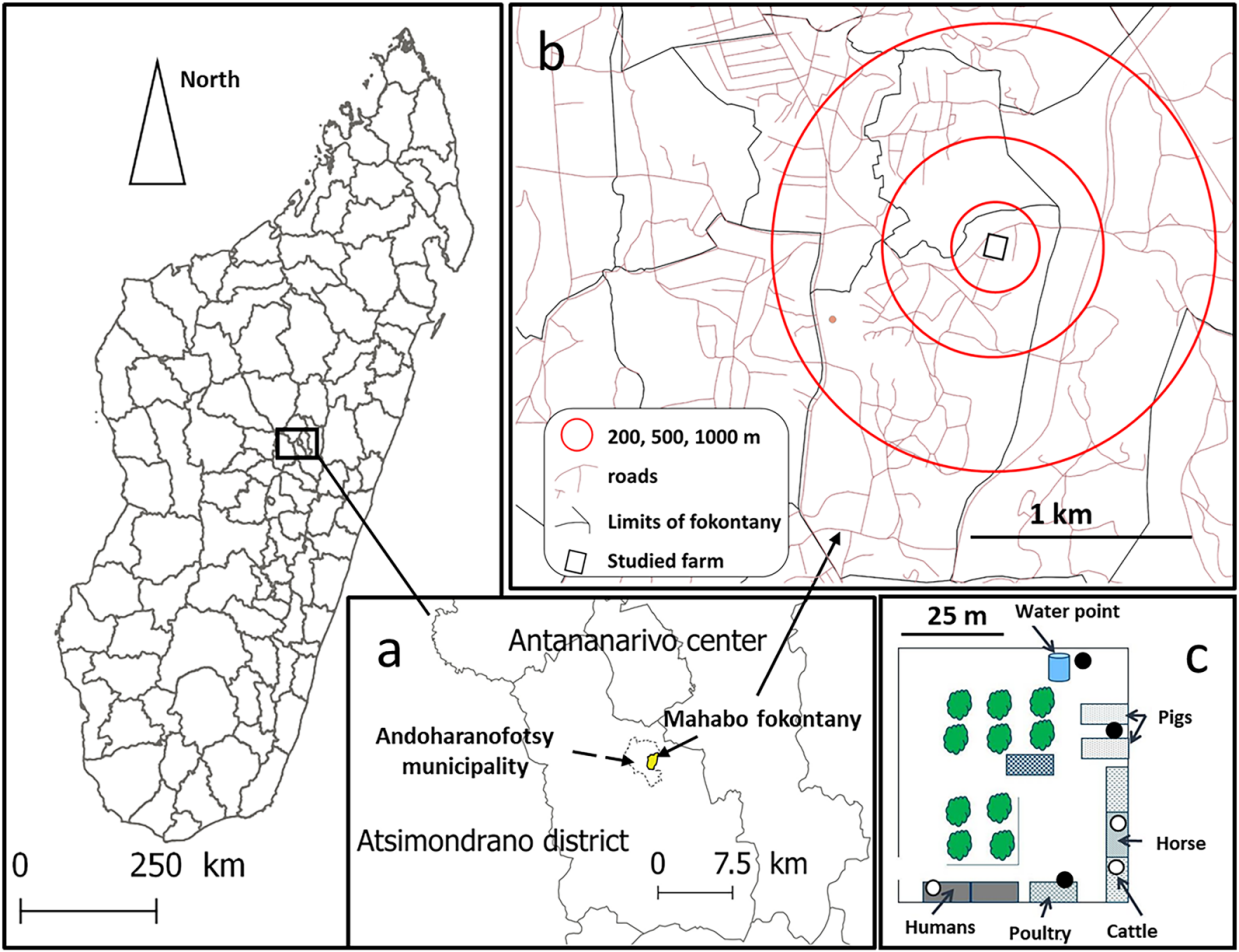
Location of the farm, Andoharanofotsy municipality, district of Atsimondrano (Madagascar). **a** Location of Fokontany Mahabo (bounded by the continuous black line). **b** Location of the farm (delimited by the black rectangle). The red circles denote the 200-m, 500-m and 1-km buffers from which the NDVI and NDWI were extracted. **c** Location of the light traps (filled circle, outdoor traps; open circle, indoor traps). NDVI, Normalised Difference Vegetation Index; NDWI, Normalised Difference Water Index

Our study was performed on a peri-urban backyard farm (18°58′45–55″S, 47°32′20–30″E), in Mahabo fokontany (1258 m a.s.l.), Andoharanofotsy municipality (approx. 7 km south of the Antananarivo centre) (Fig. 1a, b). This farm was selected because it is a small backyard farm, which is the predominant type of farm in peri-urban Antananarivo, and it was easily accessible. This farm hosts humans, horses, cattle, poultry, dogs, pigs and rabbits, and this wide variety of potential hosts increased the chances of collecting a large diversity of vector species with different feeding preferences. It consists of a small concrete house and animal shelters surrounded by residential houses, large areas of rice paddies and watercress irrigated by a canal and open areas of herbaceous savannah where cattle can graze [24, 25].

Mosquitoes were collected alive using six CDC miniature light traps (LTs) (BioQuip Products, Inc, Rancho Dominguez, CA, USA), one placed at each of six location. Three traps were placed indoors: one in the house (LTHu), one in the horse shelter (LTHo) and one in the cattle shelter (LTCa). The other three traps were placed outdoors: one near the pig enclosure (LTPi), one near the poultry park (LTPo) and one near a water point (LTWp) (Fig. 1c), at a distance of 1 to 2 m from hosts. One night of capture (from 5  p.m. to 8 a.m. the next day) was carried out every 15 days from 12 January 2017 to 26 April 2018.

Collected mosquitoes were transported to the laboratory at the Institut Pasteur de Madagascar, in Antananarivo, where they were killed with chloroform vapor and identified using the keys of Ravaonjanahary [26] for *Aedes*, Grjebine [27] for *Anopheles*, Doucet [28] for *Coquillettidia,* Edwards [29] for *Culex* and da Cunha Ramos [30] for *Uranotaenia*.

### Moon, climatic and environmental data extraction

Daily records of meteorological parameters (precipitation, temperature and relative humidity [RH]) were obtained from NASA Langley Research Center (LaRC) [31]. Daily percentage of moon illumination [MI] was obtained from the Time and Date AS Company (Stavanger, Norway) [32]. Bi-weekly Normalised Difference Vegetation Index (NDVI) (minimum, mean and maximum values) and Normalised Difference Water Index (NDWI) data for the area within a 200-m, 500-m and 1-km radius buffer surrounding the farm were downloaded from the Sen2Extract web application [33]. All of these climatic, environmental and MI data were extracted from October 2016 to April 2018.

### Statistical analysis

Statistical analyses were carried out in R software version 4.2.2 [34]. The Shannon (H) and Simpson (S) diversity indices were used to compare diversity between traps. The non-parametric estimator Chao1 [35] and abundance-based coverage estimator (ACE) [36] were used to estimate the true species richness of mosquito communities [37, 38]. To determine whether the number of collected mosquitoes reach the point at which the species richness is saturated, we constructed rarefaction curves using the rarecurve function from the 'Vegan' package [39].

Differences in the species composition between mosquitoes captured in the six traps over the 16 months of collection were analysed with the non-metric Multi-Dimensional Scaling (nMDS) ordination program [40]. Analysis of similarities (ANOSIM) was used to test the statistical significance of the MDS analysis. This method also estimates stress, which is an index aggregating representation errors, with stress values near zero being the best. Mosquito abundances in each of the six traps were compared using a Kruskal–Wallis H-test, followed by Dunn’s multiple comparisons post hoc test to determine which pairs of location of light traps were different.

The environmental variables used were the rescaled (initial values were divided by 100) mean value of the NDVI and NDWI extracted from the most recent Sentinel-2 image on 1–3 days before the sampling date. Average temperature, average RH and accumulated precipitation variables were calculated for the following 22 lag periods: 1, 1–2, 1–3, 1–4 (1 month) and 2, 2–3, 3, 3–4, 4, 4–5, 5–6, 6–7, 7–8, 8–9, 9–10, 10–11 and 11–12 weeks before the sampling periods, and 2, 3, 1–2, 1–3 and 2–3 months before the sampling periods. This time range covers the adult mosquito diapause period (3 months) [41], which probably affects the seasonal dynamics of mosquito abundance [42].

Models explaining the indoor and outdoor density of each of the four most abundant species were developed. Data from indoor and outdoor locations were analysed separately as it was suspected that some variables, such as MI, differentially impact outdoor and indoor abundance [43]. Models were developed in five steps, with one step to develop univariable models and four steps to develop multivariable models to identify the best fit model explaining the indoor and outdoor density of each of the four most abundant species.

- Step 1: A univariable model was created using the indoor and outdoor mosquito densities (average number of mosquitos per trap and per capture session) of each of the four most abundant species as the response variable. Moon illumination, environmental (NDVI and NDWI from the 200-m, 500-m and 1-km buffer areas) and climatic variables from the 22 lag periods were included as explanatory variables. Because data were not distributed normally and overdispersion with zero-inflation were detected with the *DHARMa* package [44] in the univariates Poisson models, a univariate model was created using the glm.nb function.
- Step 2: Two generalised linear models (GLMs) using the glm function for Poisson distribution and the glm.nb function for negative binomial distribution were constructed. Moon illumination + the environmental variables for one of the three buffer areas and the variable (either temperature, precipitation or RH) of the 22 lag periods that exhibited the lowest corrected Akaike information criterion (AICc) [45] value in the univariable model were retained as covariates in these two GLMs.
- Step 3: The *DHARMa* package [44] was used to test the presence of overdispersion or zero-inflation in these two subsequent GLMs (Poisson and negative binomial [NB]) by simulating their scaled residuals with the simulateResiduals function in R. The model without overdispersion (< 1) and zero-inflation (< 1) was retained, and the model with the smallest AIC and Bayesian information criterion (BIC) values was retained according to Liaqat et al. [46]. When overdispersion and zero-inflation were detected in both the Poisson and NB models, four subsequent models, namely, the zero-inflated Poisson (ZIP), zero-inflated negative binomial (ZINB), hurdle–Poisson (HP) and negative binomial hurdle (NBH) models, were applied with the same covariates. The model with the smallest AIC and BIC values was retained, according to Liaqat et al. [46].
- Step 4: The variance inflation factor (VIF) of covariates of the model which better fit the data was compared using the check_collinearity function from the performance package [47]. By excluding covariates with the highest VIF (> 10) values, the dredge function from R package *MuMIn* [48] was run to output all possible combinations of covariates to build a final model.
- Step 5: Finally, the Hosmer–Lemeshow goodness-of-fit test was assessed using the hoslem.test function (*ResourceSelection* packages) [49].

The final model was used to calculate the incidence rate ratio (IRR) for the four most abundant species densities indoors and outdoors. The predicted indoor and outdoor densities were derived from the corresponding final model using the predict function.

## Results

From 12 January 2017 to 26 April 2018, a total of 46,737 mosquitoes were collected in 189 trap-nights, corresponding to a mean of 247 mosquitoes (standard deviation [SD] 441.39) collected per LT (Table 1). At least 20 mosquito species belonging to seven genera (*Aedes*,* Anopheles*,* Coquillettidia*,* Culex*,* Lutzia*,* Mansonia* and *Uranotaenia)* were collected.

**Table 1:** Mosquito species collected in light traps at six locations on the Mahabo farm, Andoharanofotsy, Madagascar, from January 2017 to April 2018

Mosquito species^a^	Trap locations^b^ and collection statistics^c^																												
	LTHo (32)^d^				LTHu (32)				LTWP (32)				LTPi (32)				LTPo (30)				LTCa (31)				All light traps (189)				
	*n*	Pos %	Mean	SD	*n*	Pos %	Mean	SD	*n*	Pos %	Mean	SD	*n*	Pos %	Mean	SD	*n*	Pos %	Mean	SD	*n*	Pos %	Mean	SD	*n*	Pos %	Mean	SD	% spp.
*Ae. albopictus*	0	0.0	0.00	0.00	0	0.0	0.00	0.00	0	0.0	0.00	0.00	1	3.13	0.03	0.18	0	0.0	0.00	0.00	0	0.0	0.00	0.00	1	0.53	0.01	0.07	0.00
*Ae. argenteopunctatus *^*v,f*^	0	0.0	0.00	0.00	0	0.0	0.00	0.00	0	0.0	0.00	0.00	0	0.0	0.00	0.00	1	3.33	0.03	0.18	1	3.23	0.03	0.18	2	1.06	0.01	0.10	0.00
*Ae. fowleri *^*v,f*^	1	3.13	0.03	0.18	0	0.0	0.00	0.00	0	0.0	0.00	0.00	4	3.13	0.13	0.71	0	0.0	0.00	0.00	0	0.0	0.00	0.00	5	1.06	0.03	0.30	0.01
*Ae.* sp. ^e^	0	0.0	0.00	0.00	0	0.0	0.00	0.00	1	3.13	0.03	0.18	0	0.0	0.00	0.00	0	0.0	0.00	0.00	0	0.0	0.00	0.00	1	0.53	0.01	0.07	0.00
*An. coustani*	192	75.0	6.00	9.78	8	18.75	0.25	0.62	42	31.25	1.31	3.46	284	56.25	8.88	13.07	73	23.33	2.43	3.74	456	83.87	14.71	26.57	1055	53.44	5.58	13.69	2.26
*An. gambiae* s.l.	642	84.38	20.06	25.46	42	46.88	1.31	2.01	19	25.0	0.59	1.33	33	28.13	1.03	2.88	17	26.67	0.57	1.52	343	74.19	11.06	18.08	1096	47.62	5.80	14.80	2.35
*An. rufipes *^*v,p*^	2	6.25	0.06	0.25	0	0.0	0.00	0.00	0	0.0	0.00	0.00	1	3.13	0.03	0.18	1	3.33	0.03	0.18	0	0.0	0.00	0.00	4	2.12	0.02	0.15	0.01
*An. squamosus/cydippis*	88	53.13	2.75	4.55	2	6.25	0.06	0.25	0	0.0	0.00	0.00	97	43.75	3.03	10.09	3	6.67	0.10	0.40	96	45.16	3.10	5.93	286	25.93	1.51	5.32	0.61
*An.* sp.	0	0.0	0.00	0.00	0	0.0	0.00	0.00	0	0.0	0.00	0.00	0	0.0	0.00	0.00	0	0.0	0.00	0.00	1	0.0	0.03	0.00	1	0.53	0.01	0.07	0.00
*Cq. grandidieri*	125	59.38	3.91	8.31	0	0.0	0.00	0.00	10	28.1	0.31	0.55	90	53.13	2.81	5.41	11	23.33	0.37	0.76	60	48.39	1.94	5.50	296	35.45	1.57	4.85	0.63
*Cq. rochei*	1	3.13	0.03	0.18	0	0.0	0.00	0.00	1	3.13	0.03	0.18	2	3.13	0.06	0.35	0	0.0	0.00	0.00	0	0.0	0.00	0.00	4	1.59	0.02	0.18	0.01
*Cx. antennatus*	7237	100.0	226.16	279.36	37	43.75	1.16	2.08	643	45.0	20.09	37.23	11,661	93.75	364.41	728.19	825	76.67	27.50	31.81	11,821	93.55	381.32	486.90	32,224	79.37	170.50	407.70	68.95
*Cx. decens*	1	3.13	0.03	0.18	0	0.0	0.00	0.00	0	0.0	0.00	0.00	0	0.0	0.00	0.00	0	0.0	0.00	0.00	0	0.0	0.00	0.00	1	0.53	0.01	0.07	0.00
*Cx. giganteus*	11	15.63	0.34	1.00	1	3.13	0.03	0.18	12	28.1	0.38	0.72	24	21.88	0.75	1.78	9	23.33	0.30	0.60	6	16.13	0.19	0.48	63	17.99	0.33	0.96	0.13
*Cx. pipiens*	0	0.0	0.00	34.14	12	6.25	0.38	1.95	5	9.38	0.16	0.53	0	0.0	0.00	0.00	9	13.33	0.30	0.95	0	0.0	0.00	14.82	26	5.29	0.14	0.092	0.06
*Cx. poicilipes *^*v,f,p*^	786	90.63	24.56	34.14	4	12.50	0.13	0.34	91	56.25	2.84	4.82	335	71.88	10.47	17.78	167	76.67	5.57	7.07	351	74.19	11.32	14.82	1734	63.49	9.17	18.92	3.71
*Cx. quinquefasciatus*	1180	87.50	36.88	31.18	3583	93.75	111.97	88.22	274	68.75	8.56	11.81	298	81.25	9.31	10.09	598	86.67	19.93	40.49	3327	90.32	107.32	122.39	9260	84.66	48.99	78.15	19.81
*Cx. tritaeniorhynchus*	1	3.13	0.03	0.18	0	0.0	0.00	0.00	3	3.13	0.09	0.53	1	3.13	0.03	0.18	0	0.0	0.00	0.00	0	0.0	0.00	0.00	5	1.59	0.03	0.24	0.01
*Cx.* sp.	1	3.13	0.03	0.00	0	0.0	0.00	0.00	1	3.13	0.03	0.00	0	0.0	0.00	0.00	1	3.33	0.03	0.00	0	0.0	0.00	0.00	3	1.59	0.02	0.13	0.01
*Cx. univittatus*	17	21.88	0.53	1.11	15	6.25	0.47	2.05	7	9.38	0.22	0.91	12	25.00	0.38	0.71	6	16.67	0.20	0.48	4	9.68	0.13	0.43	61	14.81	0.32	1.10	0.13
*Lt. tigripes *^*v*^	0	0.0	0.00	0.00	0	0.0	0.00	0.00	6	6.2	0.19	0.78	1	3.13	0.03	0.18	0	0.0	0.00	0.00	0	0.0	0.00	0.00	7	1.59	0.04	0.33	0.01
*Ma. uniformis*	124	62.50	3.88	7.20	35	50.00	1.09	1.53	39	50.00	1.22	1.86	118	71.88	3.69	4.92	27	36.67	0.90	1.42	244	67.74	7.87	11.22	587	56.61	3.11	6.17	1.26
*Ur. alboabdominalis*	0	0.0	0.00	0.00	0	0.0	0.00	0.00	5	9.38	0.16	0.53	0	0.0	0.00	0.00	0	0.0	0.00	0.00	1	3.23	0.03	0.18	6	2.12	0.03	0.23	0.01
*Ur.* sp.	0	0.0	0.00	0.00	0	0.0	0.00	0.00	5	3.13	0.16	0.00	1	3.13	0.03	0.00	3	3.33	0.10	0.00	0	0.0	0.00	0.00	9	2.65	0.05	0.33	0.02
Total* n* mosquitoes	10,409				3739				1164				12,963				1751				16,711				46,737				
Total* n* species	15				10				16				17				14				12				20				

This table lists the some of the mosquito species found to be naturally infected with pathogens in Madagascar (in Additional file 1: Table S1) and the mosquito species naturally infected by worms (^f^), *Plasmodium* parasites (^p^) and arbovirus (^v^) in other countries

*Ca* Cattle,* Ho* horse,* Hu* house/humans,* LT* light traps,* Pi* pigs,* Po *poultry,* SD* standard deviation,* WP* water point

^a^Mosquito species:* Ae.*, *Aedes*;* An.*, *Anopheles*;* Cq.*, *Coquillettidia*;* Cx.*, *Culex*;* Ur.*,* Lt.*, Lutzia; Ma. Mansonia; *Ur. Uranotaenia*

^b^Indoor traps were located in the house (LTHu), in the horse shelter (LTHo), in the cattle shelter (LTCa). Outdoor traps were located near the pig enclosure (LTPi), near the poultry park (LTPo) and near a water point (LTWp)

^c^*n*, Cumulative number of individuals in all capture sessions; Pos %, percentage of positive traps (traps where the species is present); % spp, percentage of each species collected in Mahabo

^d^The number in parentheses following the trap location is the number of collection events

^e^*Aedes* sp. is an* Aedes* morphologically close to *Aedes mathioti*

**Fig. 2:**
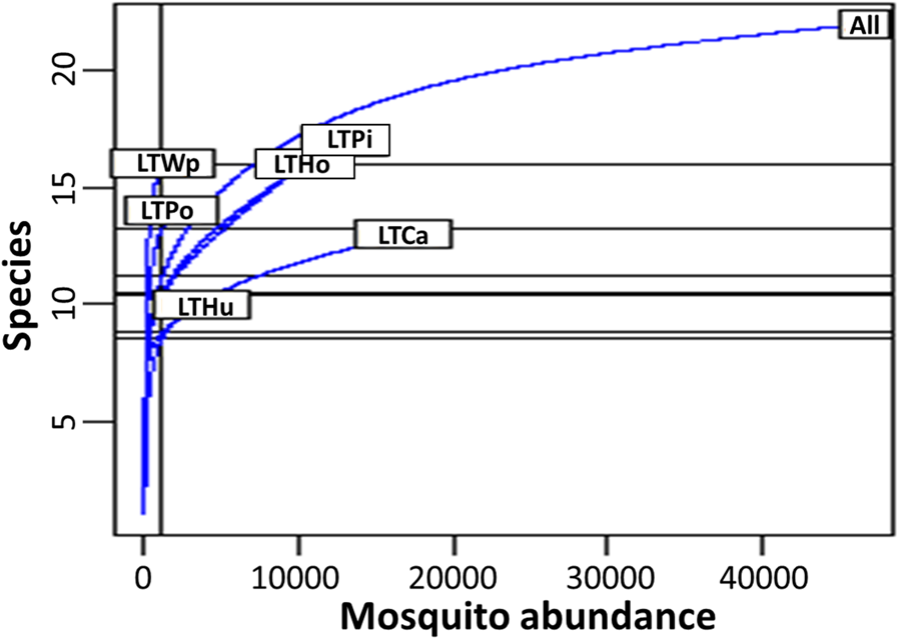
Rarefaction curves representing species richness of mosquitoes collected in traps at six locations on the Mahabo farm, Andoharanofotsy, Madagascar, from January 2017 to April 2018. Indoor traps were placed in the house (LTHu), horse shelter (LTHo) and the cattle shelter (LTCa); outdoor traps were placed near the pig enclosure (LTPi), near the poultry park (LTPo) and near a water point (LTWp). Ca, Cattle; Ho, horse; Hu, humans; LT, light traps; Pi, pigs; Po, poultry; WP, water point

For the LTCa, the rarefaction curve (plot of the number of species against the number of collections) stabilised at 12 species (Fig. 2). The greatest number of species (16) was obtained in the LTPi and the smallest (10) in the LTHu. The rarefaction curves observed for the remaining traps indicated that an additional trapping effort was needed to capture all the diversity present, in particular in the LTWp and LTPo.

The S_Chao1_ and S_ACE_ estimated that the highest number of species would be expected in the LTHo (25 species) and LTPi (24 species), and the lowest number of species would be observed in the LTHu (10 species). The Shannon and Simpson indices were higher in both the LTWp and LTPo, indicating that mosquitoes were distributed more equitably in these places. Both indices were lower in the LTHu (Additional file 2: Table S2).

**Fig. 3:**
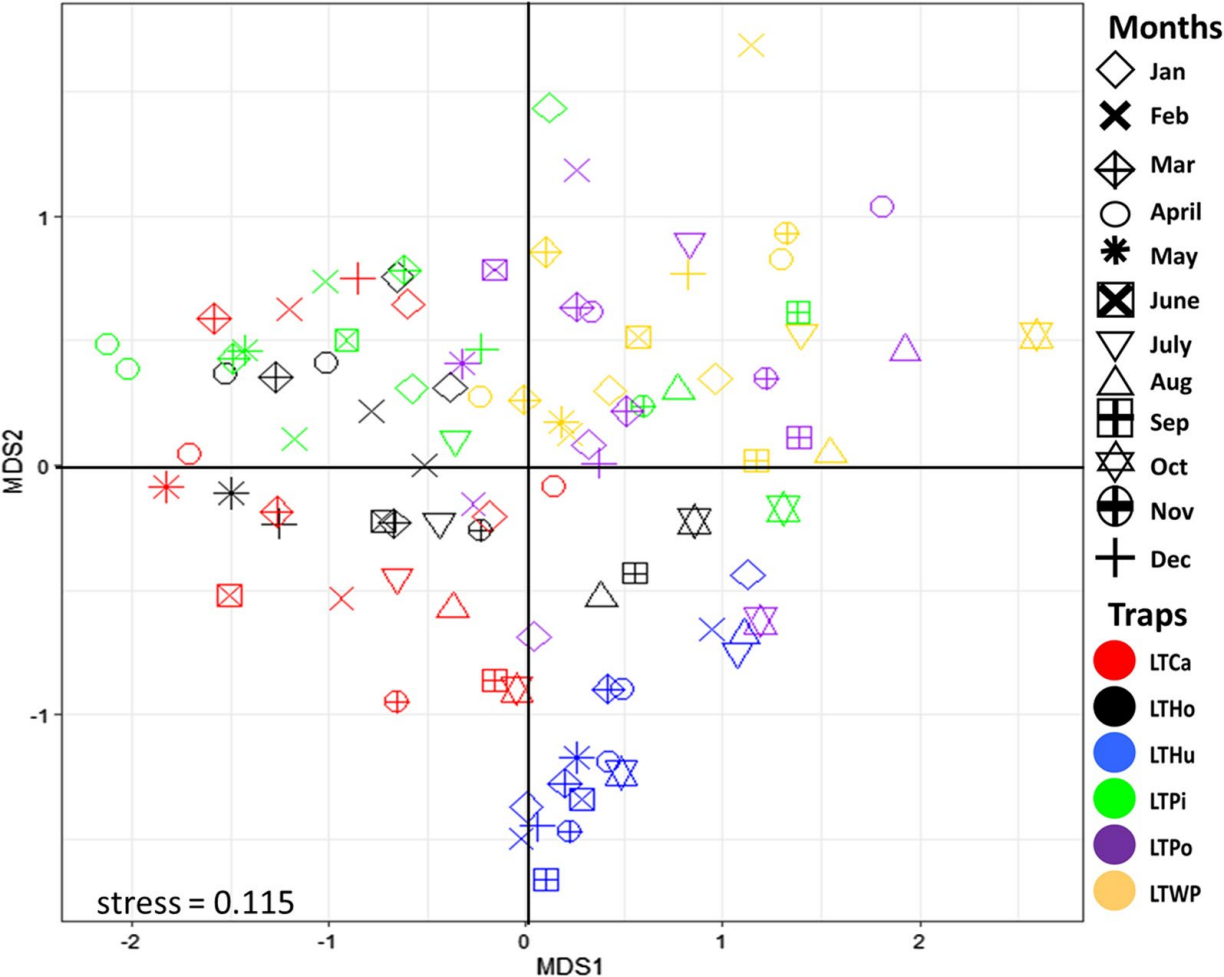
Nonmetric multidimensional scaling (MDS) ordination showing the differences in assemblages of mosquitoes caught in traps at the six different locations on the Mahabo farm, Andoharanofotsy, Madagascar, indicated on figure by different colours, and at different months of the year, indicated by different symbols, from January 2017 to April 2018. LTCa, LTHo, LTHu, Indoor traps in the house, horse shelter and cattle shelter, respectively; LTPi, LTPo, LTWp, outdoor traps near the pig enclosure, the poultry park and a water point, respectively

The species composition differed between the six trap locations during the 16 months of the study (ANOSIM statistic, *R* = 0.3912, *P* = 1e−04) (Fig. 3). The stress value of 0.115 reflects that the differences between the actual distances and their predicted values is slightly fair. The assemblage collected in the LTHu was distinctly separated from those of the other assemblages. The assemblage collected in indoor LTs (LTCa, LTHo, LTHu) were separated from those collected outdoors (LTPi, LTPo, LTWp) on the second axis.

Mosquitoes of *Culex* genus, comprising at least eight species, were dominant, accounting for 92.8% of the total number of mosquitoes collected, followed by those of *Anopheles* genus, comprising at least four species (5.2% of total collected mosquitoes) (Table 1). The remaining 2.0% consisted of mosquitoes of genus *Aedes* (4 species), *Coquillettidia* (2 species), genus *Lutzia* (1 species), genus *Mansonia* (1 species) and genus *Uranotaenia* genus (at least 1 species). *Culex antennatus* (68.9% of individuals) was by far the most abundant species, followed by *Cx. quinquefasciatus* (19.8%), *Cx. poicilipes* (3.7%) and *An. gambiae* s.l. (2.3%). Eight species (*Cx. antennatus, Anopheles coustani, An. gambiae* s.l.,* Cx. giganteus*,* Cx. poicilipes*,* Cx. quinquefasciatus*,* Mansonia uniformis* and *Cx. univittatus)* were collected in all six LTs but their abundance varied between LTs and time periods. The largest number of mosquitoes was collected in LTCa and the smallest in LTWp (Kruskal–Wallis H-test, *H* = 46.99, *df* = 5, *P* < 0.001) (Additional file 3: Table S3). Dunn’s post hoc tests showed that three-, five- and sixfold more mosquitoes were collected in the LTCa compared to the LTHu (*α* = 0.001), LTPo (*α* = 0.000) and LTWp (*α* = 0.001), respectively. Regarding outdoor LTs, the LTPi provided three- to fourfold more mosquitoes than the LTPo (*α* = 0.002) and LTWp (*α* = 0.001); regarding indoor LTs, the LTHu provided threefold more mosquitoes than the LTWp (*α* = 0.006), and the LTHo provided twofold more mosquitoes than the LTHu (*α* = 0.019).

Ranking of the 20 mosquito species showed that *Cx. antennatus* was the dominant mosquito species in the LTPi, LTCa, LTHo, LTWp and LTPo, with relative abundances of 90.0%, 71.0%, 69.5%, 55.2% and 47.1%, respectively (Additional file 4: Fig. S1). In the latter four traps, the second ranking was occupied by *Cx. quinquefasciatus*, with relative abundances of 34.0% (LTPo), 23.5% (LTWp), 20.0% (LTCa) and 11.0% (LTHo). *Culex quinquefasciatus* occupied the first ranking only for the LTHu, inside the house, where it accounted for 96.0% of mosquitoes collected; *An. gambiae* s.l. was the second most abundant species in the LTHu, but represented only 1.0% of individuals collected. In LTPi, the second most abundant species was *Cx. poicilipes* (2.6%), closely followed by *Cx. quinquefasciatus* (2.3%) and *An. coustani* (2.2%).

**Fig. 4:**
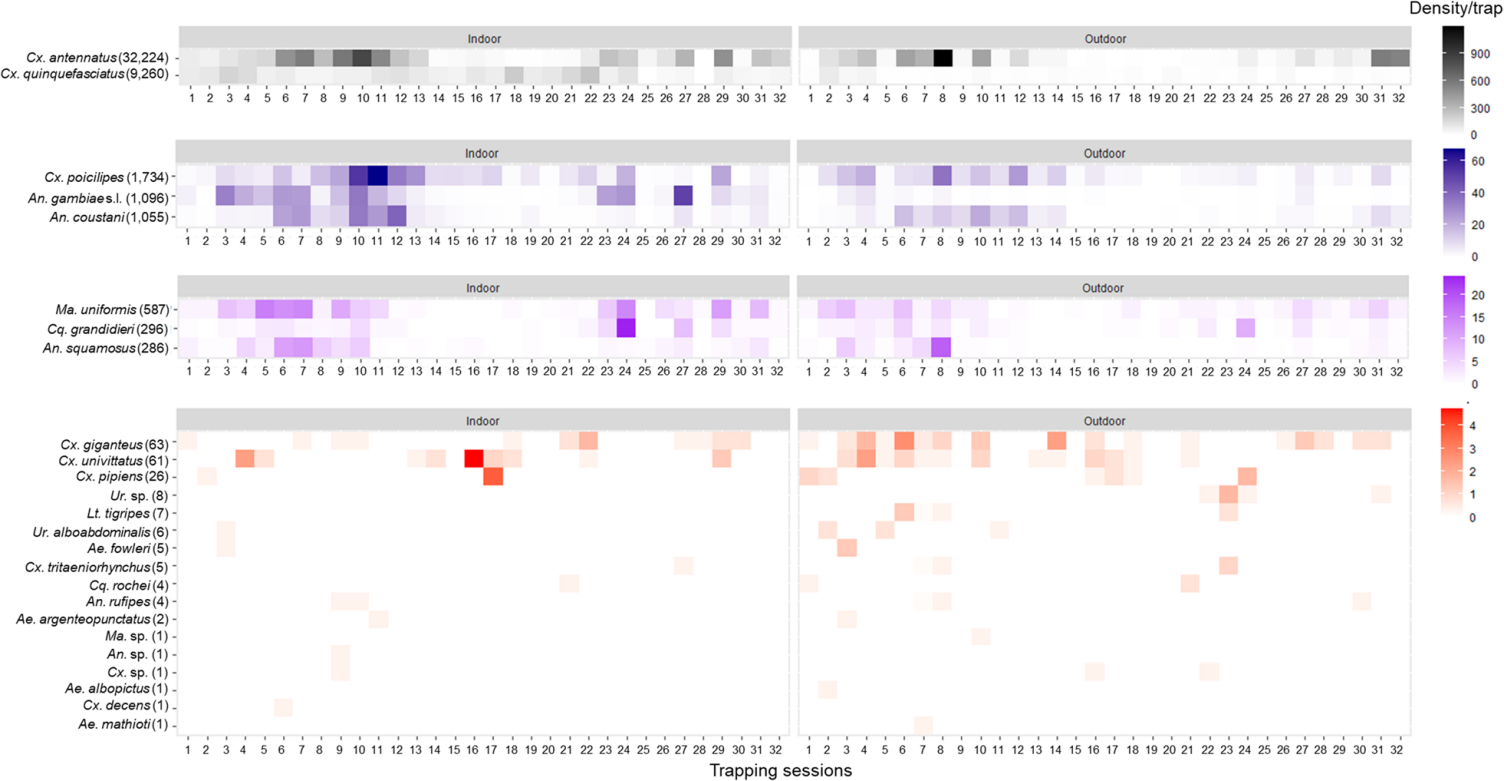
Variations in the monthly density of mosquitoes collected in the light traps located in indoor and outdoor environments on the Mahabo farm, Andoharanofotsy, Madagascar, from January 2017 to April 2018

The abundance of specimens collected varied according to the month of capture (Kruskal–Wallis H-test, *H* = 24.92, *df* = 11, *P* < 0.01). The abundance of *Ma. uniformis*,* Cx. antennatus*,* An. squamosus*/*cydippis*,* Cx. poicilipes*, *An. gambiae* s.l. and *An. squamosus* started to increase in February, with the highest abundance of *An. gambiae* s.l. observed in February, of *Ma. uniformis* in March, of *Cx. antennatus* and *An. squamosus/cydippis* in April and of *Cx. poicilipes* in June (Fig. 4). The abundance of these species decreased greatly after their respective highest peak period, and mosquitoes were rarely collected in the cold dry season (between August and November). *Culex quinquefasciatus* was abundant throughout the year, with four abundance peaks observed in February, June, September and November. *Anopheles coustani* was mostly abundant during the wet season, when four abundance peaks were observed (from December to May), and rarely collected between August and October.

Indoor and outdoor abundance models for *Cx. antennatus*,* Cx. quinquefasciatus*,* Cx. poicilipes* and *An. gambiae* s.l. were further developed*.* A total of 73 covariates including MI, 66 covariates of climatic factors (22 lag periods of temperature, 22 of RH, 22 of precipitation) and six covariates of the respective three buffers of NDVI and of NDWI were assessed through univariable analysis (Additional file 5: Table S4). According to the univariable models, MI had a significant impact only on the outdoor abundance of *Cx. antennatus*,* Cx. quinquefasciatus* and *Cx. poicilipes*. Statistically significant associations were also evident between:

(i) outdoor abundance of the four species and two to 16 lag periods of temperature, and between 12 and 21 lag periods of temperature and the indoor abundance of *Cx. antennatus*,* Cx. poicilipes* and *An. gambiae*;
(ii) two to 16 lag periods of RH and outdoor abundance of the four species, four to 17 lag periods of RH and indoor abundance of these four species;
(iii) one to 11 lagged periods of precipitation and outdoor abundance of these four species, and between three and 10 lag periods of precipitation and indoor abundance of the four species;
(iv) NDVI 200-m buffer, NDVI 500-m buffer and the NDVI 1-km buffer and indoor abundance of *Cx. antennatus*, *Cx. poicilipes* and *Cx. quinquefasciatus*, respectively,
(v) the three buffers of NDWI and the outdoor and indoor *Cx. antennatus* and *An. gambiae* abundance, the indoor abundance of *Cx. quinquefasciatus* and the NDWI 500-m buffer.

Six covariates per species were used as explanatory variables (Additional file 6: Table S5) on the basis of smallest AICc and introduced in the Poisson and NB models. The Poisson model was adequate to model the outdoor abundance of only *An. gambiae* because of the absence of the overdispersion and zero-inflation (Additional file 7: Table S6). Therefore, the NB model was retained to construct the final model for outdoor and indoor densities of *Cx. antennatus*,* Cx. poicilipes* and *Anopheles gambiae* s.l. and also for the indoor abundance of *An. gambiae* s.l. due to the absence of overdispersion and zero-inflation (< 1). The outdoor abundance Poisson and NB models of *Cx. quinquefasciatus* exhibited overdispersion and were therefore not retained. Based on the lowest AIC and BIC values, NBH provided the best fit for outdoor and indoor densities of *Cx. quinquefasciatus* (Additional file 8: Table S7). Covariates with strong collinearity (VIF > 10) were excluded from the models (Additional file 6: Table S5).

The MI was retained in outdoor abundance models of *Cx. antennatus*,* Cx. quinquefasciatus* and *Cx. poicilipes*, temperature was retained in outdoor and indoor abundance models of *Cx. antennatus, Cx. poicilipes* and *An. gambiae* s.l., RH was retained only in the indoor abundance model of *Cx. antennatus*, NDWI was retained only in the outdoor abundance model of *Cx. quinquefasciatus*, precipitation was retained only in the indoor abundance model of this last species and NDVI was retained only in the outdoor abundance model of *An. gambiae* s.l. (Table 2).

**Table 2:** Model selection to estimate the factors affecting the abundance of mosquito species at Andoharanofotsy, using the corrected Akaike information criterion and weights

Mosquito species	Model	Intercept	Variables^a^	*df*	*R*^2^	logLik	AICc	Delta	Model weight	NB models (*n*)	Hosmer–Lemeshow test
*Outdoor*											
* Cx. antennatus*	NB	− 9.273	density ~ MI + Tpm3	4	75.65	− 155.975	321.4	0.00	0.231	64	*χ*^2^ = − 13.101, *P* = 1
* Cx. quinquefasciatus*	NBH	2.817	density ~ MI + NDWI 1 km	7	47.83	− 103.699	226.1	0.00	0.615	8	*χ*^2^ = − 8.84, *P* = 1
* Cx. poicilipes*	NB	− 0.013	density ~ MI + Tpm3	4	35.93	− 85.687	180.9	0.00	0.164	64	*χ*^2^ = − 1.22, *P* = 1
* An. gambiae* s.l.	Poisson	− 12.790	density ~ NDVI 0.2 km + Tpm3	3	66.39	− 29.437	65.7	0.00	0.136	64	*χ*^2^ = − 0.59 *P* = 1
*Indoor*											
* Cx. antennatus*	NB	− 15.080	density ~ Tpm3 + Rhw1	4	60.37	− 186.071	381.6	0.00	0.255	64	*χ*^2^ = − 4.588, *P* = 1
* Cx. quinquefasciatus*	NBH	4.984	density ~ Prew7–8	5	49.90	− 159.195	330.7	0.00	0.356	64	*χ*^2^ = − 2.633 *P* = 1
* Cx. poicilipes*	NB	5.10500	density ~ NDVI 0.5 km + Tpw1	4	29.77	− 106.503	222.5	0.00	0.084	64	*χ*^2^ = − 6.25, *P* = 1
* An. gambiae* s.l.	NB	− 9.226	density ~ Tpw9.10	3	43.95	− 95.823	198.5	0.00	0.163	64	*χ*^2^ = − 11.042, *P* = 1

For each species, only the first model with the lowest AICc and highest model weight is shown among the total number of computed models (NB models)

*cAIC* Second-order (corrected) Akaike’s information criterion,* Delta* difference between the current model and the minimum AICc value *df* number of variables, *loglik* canonical log-link function, * NB* negative binomial,* NBH *negative binomial hurdle, *s.l.* sensu lato

^a^MI, Moon illumination; NDVI, Normalised Difference Vegetation Index; Prem, 1- to 3-month lag period for precipitation; Prew, 1- to 12-week lag period for precipitation; Rhm, 1- to 3-month lag period for relative humidity; Rhw, 1- to 12-week lag period for relative humidity; Tpm, 1- to 3-month lag period for temperature; Tpw, 1- to 12-week lag for temperature

The variables retained in each best fit model explained approximately 75.7%, 47.8%, 35.9% and 66.4% of the outdoor densities of *Cx. antennatus*, *Cx. quinquefasciatus*, *Cx. poicilipes* and *An. gambiae* s.l., respectively. The retained variables explained approximately 60.4%, 49.9%, 29.8% and 43.9% of the outdoor densities of *Cx. antennatus*, *Cx. quinquefasciatus*, *Cx. poicilipes* and *An. gambiae* s.l., respectively (Table 2).

**Fig. 5:**
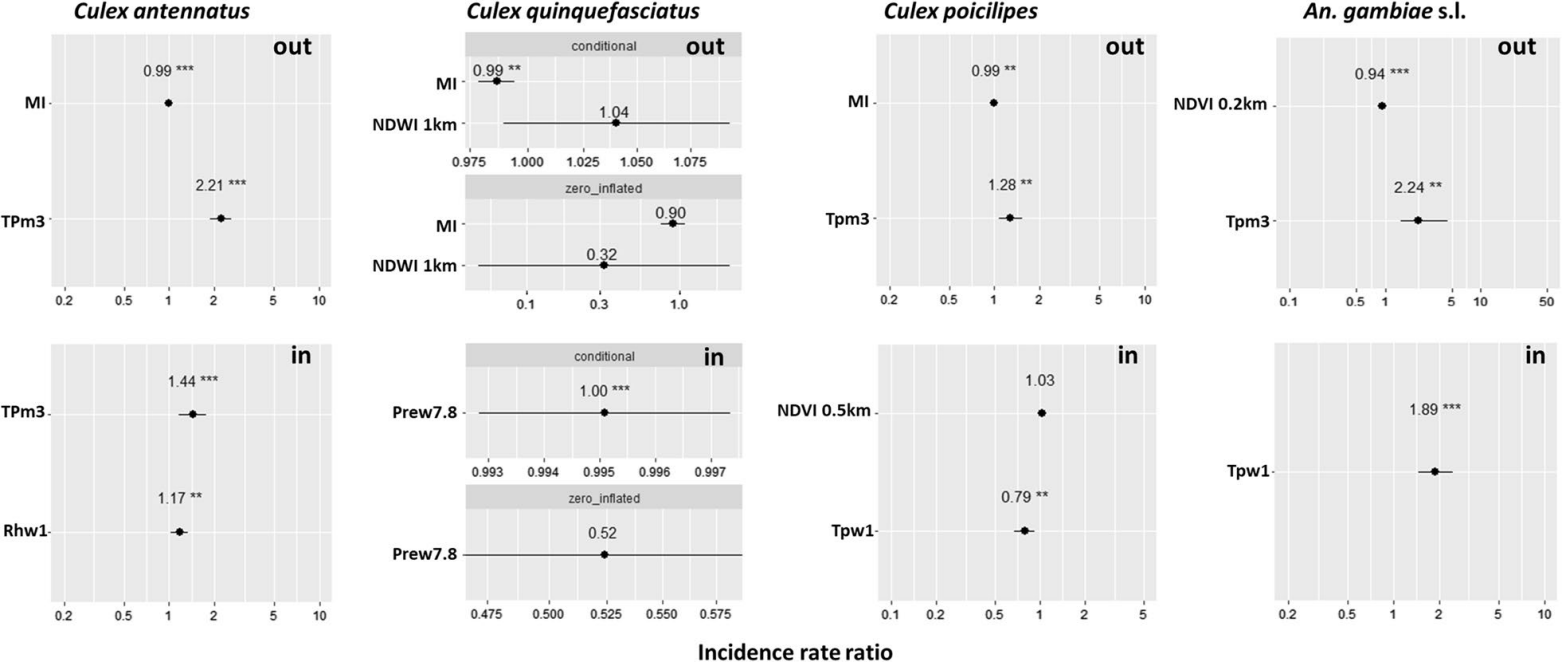
Effects of variables retained in the eight final models on the incidence rate ratio of mosquito abundance for *Cx. antennatus*, *Cx. quinquefasciatus*, *Cx. poicilipes* and *An. gambiae* s.l., with 95% confidence interval. MI, Moon illumination; NDVI, Normalised Difference Vegetation Index; Prew7.8, 7.8-week lag period for precipitation; Rhw1, 1-week lag period for relative humidity; Tpm3, 3-month lag period for temperature; Tpw1, 1-week lag period for temperature

None of the eight best fit models were rejected by the Hosmer–Lemeshow goodness-of-fit test (*P* = 1), indicating the ability of all final models to predict indoor and outdoor mosquito densities. The IRRs of the explanatory variables associated to the outdoor and indoor mosquito densities are summarised in Fig. 5 and Additional file 9: Table S8.

Moon illumination had a negative impact on outdoor mosquito abundance (IRR 0.99, 97.5% CI 0.98–1.00). This parameter was not associated with indoor abundance for any species.

Temperature was retained in the indoor and outdoor abundance models of all species except for *Cx. quinquefasciatus*. The mean temperature of the third month before the collection positively impacted outdoor abundance of *Cx. antennatus*,* Cx. poicilipes* and *An. gambiae* s.l. (1.20 < IRR < 2.25, 97.5% CI 1.07–1.88) and outdoor abundance of *Cx. antennatus* (IRR 2.13, 97.5% CI 1.17–1.76). The mean temperature of the week of the collection negatively impacted the indoor abundance of *Cx. poicilipes* (IRR 0.79, 97.5% CI 0.67–0.92) and positively impacted the indoor abundance of *An. gambiae* s.l. (IRR 1.89, 97.5% CI 1.47–2.48). Temperature (of the week preceding the collection) was negatively associated with indoor abundance of *Cx. poicilipes* (IRR 0.79, 97.5% CI 0.67–0.92).

Precipitation was positively associated with the indoor *Cx. quinquefasciatus* density (IRR 1, 97.5% CI 0.99–1) (precipitation during the 7th and 8th week before the collection).

The RH during the first week before collection was positively associated with the indoor *Cx. antennatus* abundance (IRR 1.17, 97.5% CI 1.03–1.33).

The NDVI 0.2-km buffer was negatively associated with the outdoor abundance of *An. gambiae* s.l. (IRR 0.94, 97.5% CI 0.91–0.97).

NDWI was included into the final models of *Cx. quinquefasciatus* (outdoor abundance), although the association was not statistically significant.

**Fig. 6:**
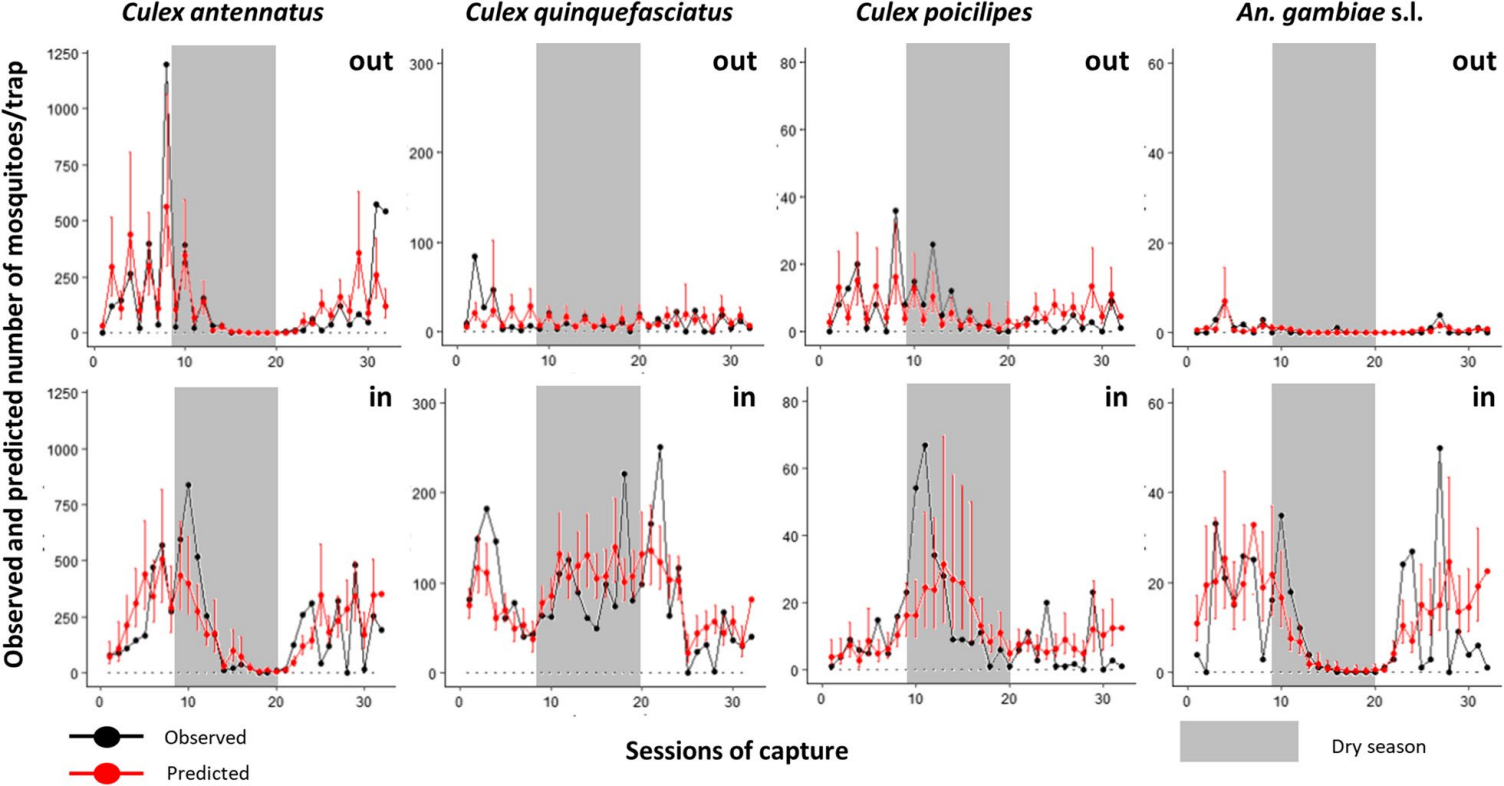
Prediction graphs of the density of the four most abundant species according to the eight final GLMs models by location of trap

The predicted and the observed mosquito densities overlapped and demonstrated that the models correctly predicted the variation of mosquito density in times for indoor and outdoor trap locations (Fig. 6).

## Discussion

### Diversity and abundance

Twenty mosquito species were identified during this study, and rarefaction curves were still increasing for most of LTs at the end of the study period (Fig. 2), suggesting that more species than those collected could have been present. Indeed, a total of 36 species have been reported in Antananarivo City and its surrounding areas so far [1, 14]. This difference is probably due to the single method (LTs) used in the present study, as well as the single collection site investigated. Applying this methodology, the greater abundance of mosquitoes collected indoors than outdoors was expected because this has already been reported in other countries [50, 51].

The abundance of *Cx. antennatus*,* Cx. quinquefasciatus*,* An. coustani* and* Ma. uniformis* has been already highlighted in Antananarivo and its surroundings [1, 14]. The abundance of *An. gambiae* s.l. and *Cx. poicilipes* is new information. The abundance of *Cx. quinquefasciatus*, a species related to peri-domestic breeding sites, and of the rice field-breeding species *Cx. antennatus*,* An. coustani*,* An. gambiae* s.l.,* Cx. poicilipes* and *Ma. uniformis* [20, 52] can be explained by the omnipresence of a mixture of waterbodies associated with both agricultural activity and poor household sanitation in Mahabo fokontany [20, 22, 24, 52]. Although *Ae. albopictus* had previously been reported to be abundant in Antananarivo [1, 53], this species was not collected in our study, probably because LTs are not suitable for collecting this diurnal species, possibly explaining its very low abundance observed here during our study period.

Our study supplements knowledge acquired during a longitudinal survey carried out on mosquito populations in Antananarivo City which dates back to the 1980s [1]. Data from that study show that although most mosquito populations declined during the cold dry season (between August and October), this decline did not represent an absence of *Cx. antennatus, An. gambiae* s.l. and *Cx. poicilipes* during this period; rather, the dynamics showed a maintenance of these species, with low numbers of individuals collected throughout the cold dry season. This seasonal variation in the populations of these three mosquito species has been reported in earlier studies using LTs and human landing catch in rural, peri-urban and urban areas of Antananarivo province [1, 52]. In contrast, *Cx. quinquefasciatus* populations were present and abundant all year round, as previously observed in Antananarivo City and its surrounding areas [1]. Because the effect of MI was suspected to be different on outdoor and indoor mosquito abundances, but the effect not very clear [43], and because other climatic variables could impact abundances differently [54], indoor and outdoor abundances were modelled separately.

### Univariable and multivariable analyses

Given the large number of covariates tested (66 lag periods of climatic variables and 3 buffers of NDVI and NDWI), AICc was used in the univariable analysis instead of AIC [55]. Our results suggest the importance of testing these different lag periods for each explanatory variable and for each species due to the differences in ecological traits associated with the four main mosquito species collected [20]. Indeed, short lag periods (weeks 1–3 prior to the collection date) of climatic conditions were found to affect the current adult populations, while those with longer lag periods affected the current larval populations or the adult populations of the previous generation [56]. Our results showed that variables with short and long lag periods were selected for inclusion in the models, demonstrating that the climatic variables impacted different mosquito generations. Moreover, outdoor climatic data were used to predict both outdoor and indoor mosquito densities, taking into account that indoor weather measurements were correlated with outdoor ones in Antananarivo City [57]. Testing three sizes of buffers for NDVI and NDWI variables showed that the best size varied per species, variable and indoor/outdoor locations.

### Drivers of mosquito abundance

After running the dredge function, which examined all possible variable combinations [48], we found that eight final models revealed for the first time that MI and climatic (temperature, RH and precipitation) and environmental factors were important drivers of mosquito abundance in at least one urbanised area of Madagascar.

One striking result was the demonstration of the effect of MI on the outdoor abundance of all four predominant mosquito species. The increase of the number of mosquitoes collected outdoors when MI decreases has been demonstrated in other countries [43, 58], with previous studies reporting the impact of MI on the length of oviposition cycles [59] and flight orientation [60]. In our study, the negative effect of MI on mosquito outdoor density could be explained by the nocturnal activity and strong positive phototropism of these mosquitoes [1, 14, 43]. MI could reduce the efficiency of LTs placed outdoors, suggesting that MI should be considered as an explanatory variable when modelling mosquito abundance using outdoor LTs.

Temperature had positive effects on mosquito densities (for *An. gambiae* s.l., *Cx. antennatus* and *Cx. poicilipes* collected, *Cx. antennatus* and *An. gambiae* s.l. collected indoor), and generally the lag period was quite important (3 months). Yet, there was one exception: temperature negatively impacted *Cx. poicilipes* indoor densities with a short lag period (1 week). Given the positive effect of long lag periods, temperature probably did not directly affect the current generation. Because increasing temperature is known to increase host-seeking, reproduction and larval development [61], an increase in temperature 3 months prior to the collection probably affected the previous generation and led to an increased adult density of the current one. Positive relationships between monthly antecedent temperature and mosquito abundance have been observed in other countries [62].

The positive correlation between first-week lagged RH and indoor *Cx. antennatus* densities might be explained by the fact that increases in RH enhance the attraction of current populations to the indoor hosts. Indeed, increased RH is known to enhance the attraction of mosquitoes to warmer baits [63]. It is also possible that odorant cues increase with humidity [64]. Increasing humidity is also known to increase mosquito lifespan and abundance [61].

The positive impact of the 7- to 8-week lagged precipitation on the indoor density of *Cx. quinquefasciatus* could highlight the role of this parameter on adult populations of the previous generation, through increasing RH and larval breeding surfaces and favouring mosquito abundance [65]. Precipitation was positively associated with the densities of species for which it contributes to increases in larval breeding habitats, probably peri-domestic breeding habitats (e.g. the water point investigated in this farm).

That the NDWI was not significantly associated to at least *Cx. quinquefasciatus* density does not mean that it failed to predict mosquito abundance. The significant associations of NDVI and NDWI mainly with *Cx. antennatus*,* Cx. quinquefasciatus* and *An. gambiae* s.l. densities resulting from the univariable analysis highlights the suitability of these parameters to predict mosquito abundance [61, 66]. The role of the NDVI 0.2-km buffer in driving mosquito abundance (i.e. *An. gambiae* s.l.) might be related to the role of plants as nectar sources [67] and/or to the presence of aquatic plants which stimulate mosquito oviposition [68] and/or to the presence of terrestrial plants that provide humidity favourable for outdoor-resting mosquitoes [69, 70]. The NDWI, a parameter used to identify open water, might reflect the presence of larval breeding habitats such as rice fields and canals present in our study site [28].

Others confounding factors, such as human and animal activities, use of bednets and mosquito behaviour—all of which might affect mosquito abundance— were not assessed during this study. That only a single household was included as collection site limited our ability to describe the human and animal factors. Regarding the use of bednets as a factor that may induce vector behavioural change [71], Magbity et al. [72] demonstrated that there was insufficient evidence to show that the presence of treated nets altered the relative efficiency of LTs.

Finally, despite our data being collected at a single study site, the large number of capture sessions (*n* = 32) and trapping (*n* = 189) were performed both indoors and outdoors at that site. This large sampling effort increased the robustness of our data-based model and allowed us to characterise the drivers of the dynamics of main vector species in our study, similar to the conclusions drawn from the authors of a similar study focusing on *Culicoides* population from a single site [73]. In our study, the models from the outdoor data were able to demonstrate the bi-weekly and main seasonal patterns of mosquito densities. The models from the indoor data only demonstrated the main seasonal patterns of mosquito densities. Our results should inform mosquito control operations of public health systems at least for the peri-urban municipality level.

## Conclusions

In resource-limited contexts, longitudinal surveys carried out in a single site can be informative on mosquito dynamics and their drivers. By combining repeated sampling and using six LTs placed in close proximity to different animal hosts, we were able to capture an important diversity of mosquitoes in peri-urban areas of Antananarivo, including major and candidate vectors of important viral and parasitic pathogens. The most abundant species were *Cx. antennatus*, a major vector of WNV and RVFV, *Cx. quinquefasciatus*, a major vector of WNV, *Cx. poicilipes*, a candidate vector of RVFV and *An. gambiae* s.l., a major vector of *Plasmodium* spp. Importantly, this work shows that these four mosquito species were present all year round, although the abundance of *Cx. antennatus*,* An. gambiae* s.l. and *Cx. poicilipes* declined during the dry cold season. The main drivers of their abundance were temperature, RH and precipitation. These variables impacted mosquito densities with different lag periods, reflecting their impact on different generations of mosquitoes and different stages of their life-cycle: the previous generation and the current larval adult populations. A consistent effect of moonlight was observed on the outdoor densities of all four species, probably due to a reduction in the efficiency of LTs on moonlit nights. Multiple trapping sites should be included to increase the scope of these findings. Alternatively, another option—less resource-intensive than repeating the same longitudinal study in other sites—could be to validate the models developed here using data collected in a limited number of sites at key timepoints and assess their predictive capacities on a larger study area. Identifying the drivers of dynamics is a first step towards the development of the pathogen transmission models (R0 models) [74] that are key to informing public health stakeholders on the periods that populations are at risk for vector-borne diseases.

## Supplementary Information

**Additional file 1: Table S1**. Malagasy mosquito species in which pathogens were isolated or detected in Madagascar. GF: general feeder; H: anthropophilic species; R: rare; AL: locally abundant; MV: major vector, CV: candidate vector, PV: potential vector. WNV: West-Nile virus; RVFV: Rift-Valley fever viruse; PERV: endemic Périnet virus, BABV: Babanki virus; NgaV: Ngari virus, ANDV: Andasibe virus; BTV: Bluetongue virus; DBV: Dakar Bat virus; MgV: Mengo virus; MMP 158: unclassified virus; Ph: human* Plasmodiu*m spp; Pav: avian* Plasmodium* spp. PERV, BABV, NGAV, ANDV, DBV, MMP 158 and MgV infection natural are from Fontenille [1]. BTV infection data are from Andriamandimby et al. [2]. RVFV infection data are from Fontenille [1], Ratovonjato et al. [4] and Jeffries et al. [5]. WNV natural infection data are from Fontenille [1] and Tantely et al. [6]. Avian heamosporidia natural infection data are from Schmid et al. 2017 [7].

**Additional file 2: Table S2**. Estimated species richness and diversity of mosquito communities collected in six distinct trap locations habitats in Mahabo farm, Andoharanofotsy, Madagascar, during 16 months.

**Additional file 3: Figure S1**. Rank-abundance curve of the six light traps.** a** horse,** b** cattle,** c** poultry,** d** human,** e** pigs,** f** water point. (All field work sessions were pooled together; names of three most abundant species).

**Additional file 4: Table S3**. Comparison in the abundance of mosquitoes between six trap locations. Mosquito collections in the farm of Mahabo, Andoharanofotsy, Madagascar, from January 2017 to April 2018. LT, Light traps; Ho, horse; Hu, house/humans; WP, water point; Pi, pigs; Po, poultry; Ca, cattle. In parenthesis are *Z*-values of Dunn’s test. Asterisk indicates the statistical significance of Dunn’s test.

**Additional file 5: Table S4**. Akaike information criterion corrected (AICc) from the univariate models considering the moon illumination, 22 lag periods of climatic factors and 3 buffer zones of the NDVI and NDWI. Asterisks indicate *P-value < 0.05, **P-value < 0.01 and ***P-value < 0.001.

**Additional file 6: Table S5**. Variance inflation factors and tolerance values calculated with the “check_collinearity()” function from performance” package.

**Additional file 7: Table S6**. Evaluation of the presence of overdispersion and zero inflation in Poisson and negative binomial GLMs. Asterisks indicate *P-value < 0.05, ** *P*-value < 0.01 and ***P-value < 0.001.

**Additional file 8: Table S7**. Goodness of fit for four types of regression models corresponding to the indoor and outdoor abundance of* Cx. quinquefasciatus*, with Akaike information criterion (AIC) and Bayesian information criterion (BIC) values shown as an estimate of model predictive performance. NBH, Negative binomial hurdle; PH, Poisson hurdle; ZIP, zero-inflated Poisson; ZINB, zero-inflated negative binomial.

**Additional file 9: Table S8**. Effects of variables retained in the eight final models on the Odds Rate Ratio (ORR) of the mosquito’s abundance for: Cx. antennatus, Cx. quinquefasciatus, Cx. poicilipes and An. gambiae s.l. with 95% confidence interval. MI: moon illumination, Tpw: 1–12 weeks lag for temperature; Tpm: one to three months lag for temperature; Rhw: 1–12 weeks lag for relative humidity; Rhm: 1–3 months lag for relative humidity; Prew: 1–12 weeks lag for precipitation; Prem: 1–3 months lag for precipitation. NBH: negative binomial hurdle, NB: negative binomial.

## Data Availability

All data provided and analysed during this study are included in this article.

## Abbreviations

AICc: Corrected Akaike information criterion
ANOSIM: Analysis of similarities
BIC: Bayesian information criterion
GLMs: Generalised linear models
HP: Hurdle–Poisson
IRR: Incidence rate ratio
MBDs: Mosquito-borne diseases
MI: Moon illumination
NB: Negative binomial
NBH: Negative binomial hurdle
NDVI: Normalised Difference Vegetation Index
NDWI: Normalised Difference Water Index
nMDS: Non-metric multi-dimensional scaling
PH: Poisson hurdle
RH: Relative humidity
VIF: Variance inflation factor
ZINB: Zero-inflated negative binomial
ZIP: Zero-inflated Poisson

## References

[1] Fontenille D. Arbovirus transmission cycles in Madagascar. Arch Inst Pasteur Madagascar. 1989;55:317. 2751393

[2] Andriamandimby S, Viarouge C, Ravalohery J, Reynes J, Sailleau C, Tantely M, et al. Detection in and circulation of Bluetongue virus among domestic ruminants in Madagascar. Vet Microbiol. 2015. 10.1016/j.vetmic.2015.02.009. 25736861 10.1016/j.vetmic.2015.02.009

[3] Chevalier V, Marsot M, Molia S, Rasamoelina H, Rakotondravao R, Pedrono M, et al. Serological evidence of West Nile and Usutu viruses circulation in domestic and wild birds in Wetlands of Mali and Madagascar in 2008. Int J Environ Res Public Health. 2020. 10.3390/ijerph17061998. 32197367 10.3390/ijerph17061998 PMC7142923

[4] Ratovonjato J, Olive MM, Tantely ML, Andrianaivolambo L, Tata E, Razainirina J, et al. Detection, isolation, and genetic characterisation of Rift Valley fever virus from* Anopheles* (*Anopheles*)* coustani*,* Anopheles* (*Anopheles*)* squamosus*, and* Culex* (*Culex*)* antennatus* of the Haute Matsiatra region, Madagascar. Vector Borne Zoonotic Dis. 2010;11:753–9. 10.1089/vbz.2010.0031 21028960

[5] Jeffries C, Tantely ML, Raharimalala F, Hurn E, Boyer S, Walker T. Diverse novel resident* Wolbachia* strains in Culicine mosquitoes from Madagascar. Sci Rep. 2018. 10.1038/s41598-018-35658-z. 10.1038/s41598-018-35658-z PMC6265278 30498246

[6] Tantely ML, Andriamandimby SF, Ambinintsoa MF, Raharinirina MR, Rafisandratantsoa JT, Ravalohery JP, et al. An entomological investigation during a recent Rift Valley fever epizootic/epidemic reveals new aspects of the vectorial transmission of the virus in Madagascar. Pathogens. 2024. 10.3390/pathogens13030258. 38535601 10.3390/pathogens13030258 PMC10975538

[7] Tantely ML, Cêtre-Sossah C, Rakotondranaivo T, Cardinale E, Boyer S. Population dynamics of mosquito species in a West Nile Virus endemic area in Madagascar. Parasite. 2017. 10.1051/parasite/2017005. 28134093 10.1051/parasite/2017005 PMC5780677

[8] Schmid S, Dinkel A, Mackenstedt U, Tantely ML, Randrianambinintsoa FJ, Boyer S, et al. Avian malaria on Madagascar: bird hosts and putative vector mosquitoes of different *Plasmodium* lineages. Parasit Vectors. 2017. 10.1186/s13071-016-1939-x. 28057063 10.1186/s13071-016-1939-x PMC5217334

[9] Tantely ML, Boyer S, Fontenille D. A review of mosquitoes associated with Rift Valley fever virus in Madagascar. Am J Trop Med Hyg. 2015. 10.4269/ajtmh.14-0421. 25732680 10.4269/ajtmh.14-0421 PMC4385764

[10] Tchouassi DP, Torto B, Sang R, Riginos C, Ezenwa VO. Large herbivore loss has complex effects on mosquito ecology and vector-borne disease risk. Trans Emerg Dis. 2021. 10.1111/tbed.13918. 10.1111/tbed.13918 33170555

[11] LaDeau SL, Allan BF, Leisnham PT, Levy MZ. The ecological foundations of transmission potential and vector-borne disease in urban landscapes. Funct Ecol. 2015. 10.1111/1365-2435.12487. 26549921 10.1111/1365-2435.12487 PMC4631442

[12] Lockaby G, Noori N, Morse W, Zipperer W, Kalin L, Governo R, et al. Climatic, ecological, and socioeconomic factors associated with West Nile virus incidence in Atlanta, Georgia, USA. J Vector Ecol. 2016;41:232–43. 10.1111/jvec.12218 27860011

[13] Broban A, Olive MM, Tantely ML, Dorsemans AC, Rakotomanana F, Ravalohery JP, et al. Seroprevalence of IgG antibodies directed against dengue, chikungunya and West Nile viruses and associated risk factors in Madagascar, 2011 to 2013. Virus. 2023. 10.3390/v15081707. 10.3390/v15081707 PMC10458928 37632049

[14] Tantely ML, Guis H, Randriananjantenaina I, Raharinirina MR, Velonirina HJ, Cardinale E, et al. Mosquito species associated with horses in Madagascar: a review of their vector status with regard to the epidemiology of West Nile fever. Med Vet Entomol. 2021. 10.1111/mve.12544. 34427959 10.1111/mve.12544

[15] Morvan J, Rollin PE, Laventure S, Rakotoarivony I, Roux J. Rift Valley fever epizootic in the central highlands of Madagascar. Res Virol. 1992;143:407–15. 1297176 10.1016/S0923-2516(06)80134-2

[16] Domarle O, Razakandrainibe R, Rakotomalala E, Jolivet L, Randremanana R, Rakotomanana F, et al. Seroprevalence of malaria in inhabitants of the urban zone of Antananarivo, Madagascar. Malar J. 2006. 10.1186/1475-2875-5-106. 17096830 10.1186/1475-2875-5-106 PMC1654172

[17] United Nations Department of Economic and Social Affairs. World urbanization prospects: the 2018 revision. Statistical papers-United Nations (Ser. A), population and vital statistics report. United Nations: New York; 2018.

[18] Rakotomanana F, Ratovonjato J, Randremanana RV, Randrianasolo L, Raherinjafy R, Rudant JP, et al. Geographical and environmental approaches to urban malaria in Antananarivo (Madagascar). BMC Infect Dis. 2010. 10.1186/1471-2334-10-173. 20553598 10.1186/1471-2334-10-173 PMC2894838

[19] Wilke A, Benelli G, Beier JC. Anthropogenic changes and associated impacts on vector–borne diseases. Trends Parasitol. 2021. 10.1016/j.pt.2021.09.013. 34686421 10.1016/j.pt.2021.09.013

[20] Tantely ML, Le Goff G, Boyer S, Fontenille D. An updated checklist of mosquito species (Diptera: Culicidae) from Madagascar. Parasite. 2016. 10.1051/parasite/2016018. 27101839 10.1051/parasite/2016018 PMC4840257

[21] Talla C, Diallo D, Dia I, Ba Y, Ndione JA, Morse AP, et al. Modelling hotspots of the two dominant Rift Valley fever vectors (*Aedes vexans* and *Culex poicilipes*) in Barkedji, Senegal. Parasit Vectors. 2016. 10.1186/s13071-016-1399-3. 26922792 10.1186/s13071-016-1399-3 PMC4769837

[22] Baylis M, Meiswinkel R, Venter GJ. A preliminary attempt to use climate data and satellite imagery to model the abundance and distribution of *Culicoides imicola* (Diptera: Ceratopogonidae) in southern Africa. J S Afr Vet Assoc. 1999;70:80–9. 10855827 10.4102/jsava.v70i2.759

[23] San Martín JL, Brathwaite O, Zambrano B, Solórzano JO, Bouckenooghe A, Dayan GH, et al. The epidemiology of dengue in the Americas over the last three decades: a worrisome reality. Am J Trop Med Hyg. 2010. 10.4269/ajtmh.2010.09-0346. 20065008 10.4269/ajtmh.2010.09-0346 PMC2803522

[24] Defrise L. Terres agricoles face à la ville: logiques et pratiques des agriculteurs dans le maintien des espaces agricoles à Antananarivo, Madagascar. Thesis. Paris: University of Paris, AgroParisTech; 2020.

[25] Dupuy S, Defrise L, Lebourgeois V, Gaetano R, Burnod P, Tonneau JP. Analyzing urban agriculture’s contribution to a Southern City’s resilience through land cover mapping: the case of Antananarivo, capital of Madagascar. Remote Sens. 2020. 10.3390/rs12121962. 10.3390/rs12121962

[26] Ravaonjanahary C. Les *Aedes* de Madagascar (Diptera-Culicidae). Paris: O.R.S.T.O.M; 1978.

[27] Grjébine A. Insectes Diptères Culicidae Anophelinae. Paris: O.R.S.T.O.M; 1966.

[28] Doucet J. Étude des Culicidae de la région de Vangaindrano (Diptera). Paris: Mémoires de l’Institut Scientifique de Madagascar; 1951.

[29] Edwards FW. Mosquitoes of the Ethiopian Region. III. Culicine adults and pupae. London: British Museum (Natural History);1941.

[30] Da Cunha RH, Brunhes J. Insecta, Diptera, Culicidae, Uranotaenia. Paris: IRD-CIRAD; 2004.

[31] NASA Langley Research Center (LaRC) POWER project. https://power.larc.nasa.gov/data-access-viewer/. Accessed 1 Feb 2022.

[32] Time and Date AS company. Moon phases–lunar calendar for Melbourne, Victoria, Australia. https://www.timeanddate.com/astronomy/madagascar. Accessed 01 Feb 2022.

[33] Gao BC. NDWI—a Normalized Difference Water Index for remote sensing of vegetation liquid water from space. Remote Sens Environ. 1996;58:257–66. 10.1016/S0034-4257(96)00067-3

[34] R Core Team. A language and environment for statistical computing. Vienna: Foundation for Statistical Computing; 2021. https://www.R-project.org/. Accessed on 2021

[35] Colwell RK, Coddington JA. Estimating terrestrial biodiversity through extrapolation. Philos Trans R Soc Lond Ser B. Biol Sci. 1994;345:101–18 10.1098/rstb.1994.0091 7972351

[36] Magurran AE. Measuring biological diversity. Oxford: Blackwell Science; 2004.

[37] Brant HL, Ewers RM, Vythilingam I, Drakeley C, Benedick S, Mumford JD. Vertical stratification of adult mosquitoes (Diptera: Culicidae) within a tropical rainforest in Sabah, Malaysia. Malar J. 2016. 10.1186/s12936-016-1416-1. 27430261 10.1186/s12936-016-1416-1 PMC4950076

[38] Chao A, Colwell RK, Lin CW, Gotelli NJ. Sufficient sampling for asymptotic minimum species richness estimators. Ecology. 2009;90:1125–33. 19449706 10.1890/07-2147.1

[39] Oksanen J, Blanchet FG, Kindt R, Legendre P, Minchin P, O’Hara R, et al. Vegan: Community ecology package. R package version 2.0-2. 2012. https://cran.r-project.org, https://github.com/vegandevs/vegan Accessed 17 Jan 2017.

[40] Clarke K, Ainsworth M. A method of linking multivariate community structure to environmental variables. Mar Ecol Prog Ser. 1993;92:205–19. 10.3354/meps092205

[41] Robich RM, Denlinger DL. Diapause in the mosquito *Culex pipiens* evokes a metabolic switch from blood feeding to sugar gluttony. Proc Natl Acad Sci USA. 2005. 10.1073/pnas.0507958102. 16247003 10.1073/pnas.0507958102 PMC1276097

[42] Roiz D, Ruiz S, Soriguer R, Figuerola J. Climatic effects on mosquito abundance in Mediterranean wetlands. Parasit Vectors. 2014. 10.1186/1756-3305-7-333. 25030527 10.1186/1756-3305-7-333 PMC4223583

[43] Rubios-Palis Y. Influence of Moonlight on the light trap catches of the malaria vector *Anopheles nuneztovari* in Venezuela. J Am Mosq Control Assoc. 1992;8:178–80. 1431859

[44] Hartig F. DHARMa: residual diagnostics for hierarchical (multi-level/mixed) regression models. 2019. https://cran.r-project.org/web/packages/DHARMa/DHARMa.pdf. Accessed 08 Sep 2022.

[45] Burnham K, Anderson D. Model selection and multimodel inference: a practical information-theoretic approach. 2nd ed. New York: Springer; 2002.

[46] Liaqat M, Kamal S, Fischer F, Zia N. Zero-inflated and hurdle models with an application to the number of involved axillary lymph nodes in primary breast cancer. J King Saud Univ Sci. 2022;34:101932. 10.1016/j.jksus.2022.101932. 10.1016/j.jksus.2022.101932

[47] Lüdecke D, Makowski D, Waggoner P. Performance: assessment of regression models performance. R Package Version 0.4.3. 2020. https://easystats.github.io/performance/. Accessed 30 Mar 2022.

[48] Barton K. MuMIn: Multi–model inference. R package version 1.43.17. 2020. https://CRAN.R-project.org/package=MuMIn. Accessed 27 Jun 2022.

[49] Lele SR, Keim JL, Solymos P. ResourceSelection: resource selection (probability) functions for use–availability data. R package version 0.3-6. 2019. https://CRAN.R-project.org/package=ResourceSelection. Accessed 09 Jul 2023.

[50] Sriwichai P, Karl S, Samung Y, Sumruayphol S, Kiattibutr K, Payakkapol A, et al. Evaluation of CDC light traps for mosquito surveillance in a malaria endemic area on the Thai-Myanmar border. Parasit Vectors. 2015. 10.1186/s13071-015-1225-3. 26666683 10.1186/s13071-015-1225-3 PMC4678759

[51] Mwangangi J, Muturi EJ, Muriu SM, Nzovu J, Midega JT, Mbogo C. The role of *Anopheles arabiensis* and *Anopheles coustani* in indoor and outdoor malaria transmission in Taveta District, Kenya. Parasit Vectors. 2013. 10.1186/1756-3305-6-114. 23601146 10.1186/1756-3305-6-114 PMC3652741

[52] Tantely ML, Rakotoniaina JC, Andrianaivolambo L, Tata E, Razafindrasata F, Fontenille D, et al. Biology of mosquitoes that are potential vectors of Rift Valley fever virus in different biotopes of the Central Highlands of Madagascar. J Med Entomol. 2013. 10.1603/ME12069. 23802456 10.1603/ME12069

[53] Raharimalala FN, Ravaomanarivo LH, Ravelonandro P, Rafarasoa LS, Zouache K, Tran-Van V, et al. Biogeography of the two mosquito vectors, *Aedes aegypti* and *Aedes albopictus* (Diptera, Culicidae) in Madagascar. Parasit Vectors. 2012. 10.1186/1756-3305-5-56. 22433186 10.1186/1756-3305-5-56 PMC3359177

[54] Ngowo HS, Kaindia EW, Matthiopoulos J, Ferguson HM, Okumu FO. Variations in household microclimate affect outdoor-biting behaviour of malaria vectors. Well Open Res. 2017. 10.12688/wellcomeopenres.12928.1. 10.12688/wellcomeopenres.12928.1 PMC5829465 29552642

[55] Burnham KP, Anderson DR. Multimodel inference: understanding AIC and BIC in model selection. Sociol Method Res. 2004;33:261-304. 10.1177/0049124104268644.

[56] Degaetano AT. Meteorological effects on adult mosquito (*Culex*) populations in metropolitan New Jersey. Int J Biometeorol. 2005. 10.1007/s00484-004-0242-2. 15864404 10.1007/s00484-004-0242-2

[57] Pan J, Tang JJ, Caniza M, Heraud JM, Koay E, Lee HK, et al. Correlating indoor and outdoor temperature and humidity in a sample of buildings in tropical climates. Indoor Air. 2021. 10.1111/ina.12876. 34138487 10.1111/ina.12876

[58] Guimarães AE, Gentile C, Lopes CM, de Mello RP. Ecology of mosquitoes (Diptera: Culicidae) in areas of Serra do Mar State Park, State of São Paulo, Brazil. III. Daily biting rhythms and lunar cycle influence. Mem Inst Oswaldo Cruz. 2000. 10.1590/S0074-02762000000600002. 10.1590/s0074-02762000000600002 11080757

[59] Kampango A, Cuamba N, Charlwood JD. Does moonlight influence the biting behaviour of *Anopheles funestus*? Med Vet Entomol. 2011. 10.1111/j.1365-2915.2010.00917.x. 21073491 10.1111/j.1365-2915.2010.00917.x

[60] Nowinszky L. Nocturnal illumination and night flying insects. Appl Ecol Environ Res. 2004;2:17–52. 10.15666/aeer/02017052

[61] Reiter P. Climate change and mosquito–borne disease. Environ Health Perspect. 2001;109:141–61. 11250812 10.1289/ehp.01109s1141 PMC1240549

[62] Reisen WK, Cayan D, Tyree M, Barker CM, Eldridge B, Dettinger M. Impact of climate variation on mosquito abundance in California. J Vector Ecol. 2007;33:89–98. 10.3376/1081-1710(2008)33[89:IOCVOM]2.0.CO;2 18697311

[63] Cribellier A, Spitzen J, Fairbairn H, Van De Geer C, Van Leeuwen JL, Muijres FT. Lure, retain, and catch malaria mosquitoes. How heat and humidity improve odour–baited trap performance. Malar J. 2020. 10.1186/s12936-020-03403-5. 33028362 10.1186/s12936-020-03403-5 PMC7542916

[64] Olanga EA, Okal MN, Mbadi PA, Kokwaro ED, Mukabana WR. Attraction of* Anopheles gambiae *to odour baits augmented with heat and moisture. Malar J. 2010. 10.1186/1475-2875-9-6. 20051143 10.1186/1475-2875-9-6 PMC2822790

[65] Mondet B, Diaïté A, Ndione JA, Fall AG, Chevalier V, Lancelot R, et al. Precipitation patterns and population dynamics of *Aedes* (*Aedimorphus*) *vexans arabiensis*, Patton 1905 (Diptera: Culicidae), a potential vector of Rift Valley Fever virus in Senegal. J Vector Ecol. 2005;3:102–6. 16007962

[66] Alahmed AM. Mosquito fauna (Diptera: Culicidae) of the eastern region of Saudi Arabia and their seasonal abundance. J King Saud Univ Sci. 2012. 10.1016/j.jksus.2010.12.001. 10.1016/j.jksus.2010.12.001

[67] Barredo E, DeGennaro M. Not just from blood: mosquito nutrient acquisition from nectar sources. Trends Parasitol. 2020. 10.1016/j.pt.2020.02.003. 32298634 10.1016/j.pt.2020.02.003

[68] Turnipseed RK, Moran PJ, Allan SA. Behavioral responses of gravid *Culex quinquefasciatus*, *Aedes aegypti*, and *Anopheles quadrimaculatus* mosquitoes to aquatic macrophyte volatiles. J Vector Ecol. 2018. 10.1111/jvec.12309. 30408300 10.1111/jvec.12309

[69] Cléments AN. The biology of mosquitoes: sensory, reception and behaviour. Wallingford: CABI Publishing. 1999;2:43.

[70] Raffy M, Tran A. On the dynamics of flying insects populations controlled by large scale information. Theor Popul Biol. 2005. 10.1016/j.tpb.2005.03.005. 16023689 10.1016/j.tpb.2005.03.005

[71] Sougoufara S, Doucouré S, Sembéne PMB, Harry M, Sokhna C. Challenges for malaria vector control in sub-Saharan Africa: resistance and behavioral adaptations in *Anopheles* populations. J Vector Borne Dis. 2017;54:4–15. 28352041 10.4103/0972-9062.203156

[72] Magbity EB, Lines JD, Marbiah MT, David K, Peterson E. How reliable are light traps in estimating biting rates of adult *Anopheles gambiae* s.l. (Diptera: Culicidae) in the presence of treated bednets? Bull Entomol Res. 2002; 10.1079/BER2001131. 10.1079/BER2001131 12020364

[73] Brugger K, Rubel F. Bluetongue disease risk assessment based on observed and projected *Culicoides obsoletus* spp. vector densities. PLoS ONE. 2013. 10.1371/journal.pone.0060330. 23560090 10.1371/journal.pone.0060330 PMC3613389

[74] Smith DL, Battle KE, Hay SI, Barker CM, Scott TW, McKenzie FE. Ross, macdonald, and a theory for the dynamics and control of mosquito-transmitted pathogens. PLoS Pathog. 2012. 10.1371/journal.ppat.1002588. 22496640 10.1371/journal.ppat.1002588 PMC3320609

