# Supplementary figures and images for: Mosquito dynamics and their drivers in peri-urban Antananarivo, Madagascar: insights from a longitudinal multi-host single-site survey
Source: Parasit Vectors. 2024 Sep 10;17:383. doi: 10.1186/s13071-024-06393-4 (PMC11385145; doi:10.1186/s13071-024-06393-4)

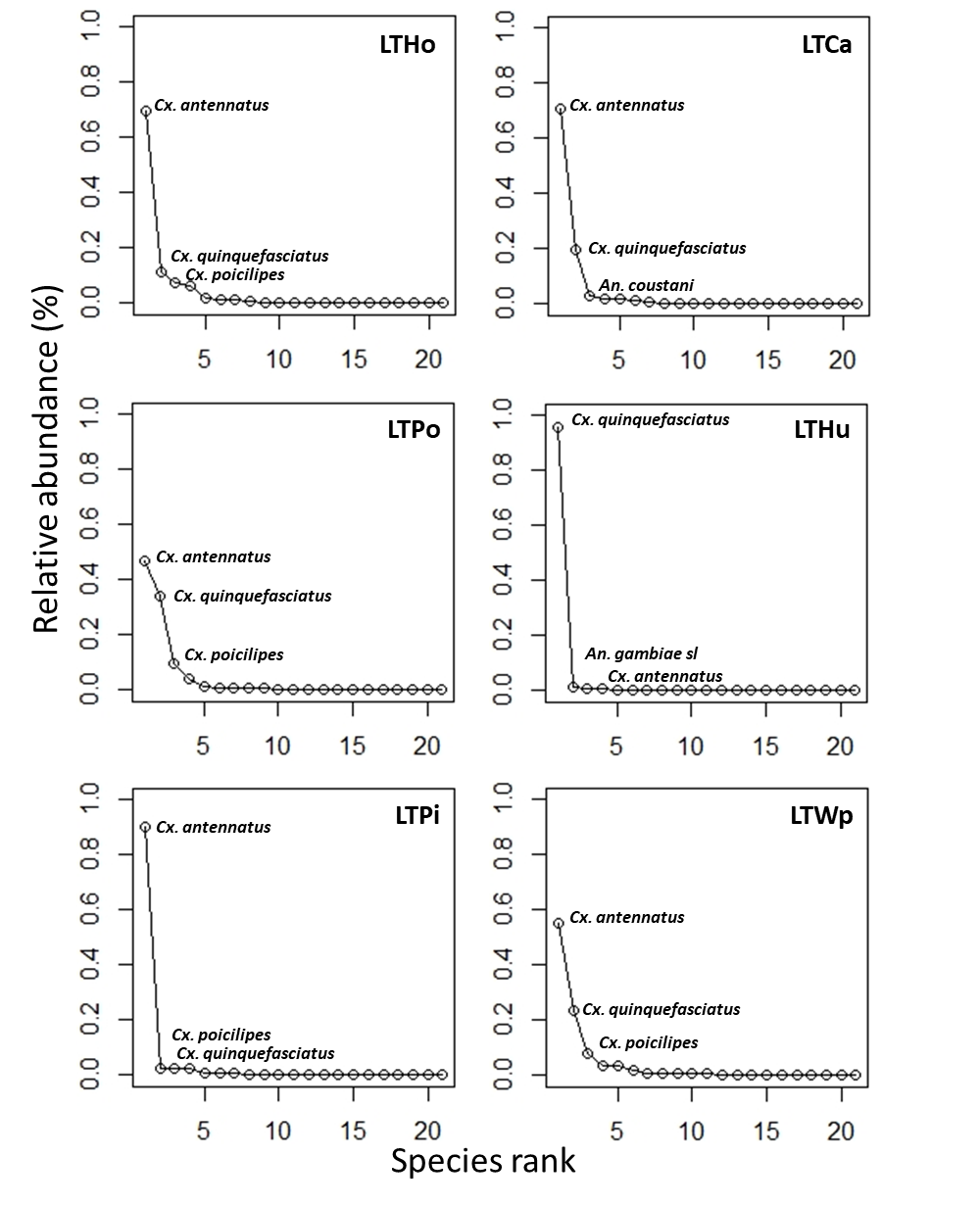

Supplement: Supplementary file 3 — Additional file 3: Figure S1. Rank-abundance curve of the six light traps. a horse, b cattle, c poultry, d human, e pigs, f water point. (All field work sessions were pooled together; names of three most abundant species). [file 13071_2024_6393_MOESM3_ESM.tiff]
